# Supplementary material for: The effectiveness of public health interventions against COVID-19: Lessons from the Singapore experience
Source: PLoS One. 2021 Mar 30;16(3):e0248742. doi: 10.1371/journal.pone.0248742 (PMC8009429; doi:10.1371/journal.pone.0248742)
Supplement: S1 File — (DOCX) [file pone.0248742.s001.docx]

**S1 File.**

**S1 Appendix:** Singapore’s approach to COVID-19

**S2 Appendix:** COVID-19 Cases in Singapore

**S3 Appendix:** Simulation results of local population

**S4 Appendix:** Simulation results migrant workers

**S5 Appendix:** Sensitivity analysis for Singapore Intervention

**S6 Appendix:** Sensitivity analysis for mitigation intervention with low isolation rate

**S7 Appendix:** Sensitivity analysis for mitigation intervention with moderate isolation rate

**S8 Appendix:** Immunity assumption

**S9 Appendix:** Uninhibited spread/herd immunity assumption

**S10 Appendix:** References

**S1 Appendix: Singapore’s approach to COVID-19**

Guided by the experience of Severe Acute Respiratory Syndrome (SARS) in 2003 which led to 228 cases and 33 deaths in Singapore, the city-state has since ramped up its infectious disease prevention measures in combating future epidemics.^1^ The relative success behind Singapore’s containment of the pandemic, leading to 25 deaths thus far, can be attributed to the government’s quick response, immediate contact tracing, targeted quarantine measures, strict quarantine management, and abundant community communication. According to a study by the Center for Communicable Disease Dynamics at Harvard University, Singapore has detected almost three times more COVID-19 cases than its neighbors as a result of its continued vigilance in contact tracing and disease surveillance.^2^

Following the identification of the outbreak in Wuhan, China, the Singaporean government acted immediately to ensure the safety of their citizens.^3^ By January 2, 2020, the government began implementing mandatory temperature screenings for all passengers on flights from Wuhan.^4^ These measures were then extended to include all passengers on flights from China on January 20, before being further extended to include temperature checking at all sea and land checkpoints on January 24.^4^ In addition, on January 22, 2020, a multi-ministry taskforce was established to coordinate the country-wide effort against COVID-19 and included preventative measures at schools and workplace, as well as business continuity plans to prevent economic disruption.^4^

Having learned from the experience of combating SARS, the Singaporean government developed physical and operational infrastructure to support rapid contact tracing, quarantine, and medical services for infected individuals. The Infectious Disease Act (IDA) was revised in 2003 in order to ensure that all measures needed to control any future outbreaks are implemented.^5^ Some of these measures include the handling and disposal of bodies due to SARS within 24 hours, active contact tracing within 24 hours of a case being identified, mandatory home quarantine enforced through technology and home visits, and thorough education of both healthcare professionals and the public.^5^ Furthermore, the IDA authorizes the Ministry of Health to serve those who disobey quarantine orders with severe punishments including large fines or imprisonment.^5^ Lastly, the IDA allows for speedy identification of each case and for the reduction of time between the onset of symptoms and isolation of the patient.^6^ The National Centre for Infectious Diseases (NCID) was established in the wake of SARS to include 330 beds, including a high-level isolation unit which treats high-risk pathogens; this has been the preferred site of care, during the early stages of the COVID-19 outbreak when the implications of infection and optimal treatment were yet to be fully understood.^7^

The initial success in containing the spread of COVID-19 has been attributed to the efficient and immediate contact tracing of patients who have been diagnosed with the virus.^8^ Although contact tracing is by no means a new innovation, Singapore’s aggressive and proactive approach, praised by the WHO, has led to the avoidance of a nation-wide spread.^9^ Upon receiving word of a newly diagnosed patient, contact tracers immediately embark on a labor-intensive attempt to identify people who have been in contact with infected individuals, thereby being able to find those who may themselves may be infected.^8^ Because the time taken for a person infected with COVID-19 to pass the infection on to others is very short, contact tracing must be swift in order to contain the outbreak.^10^ Security cameras, receipts, and work calendars are used to fill in the gaps in memory of those infected who are unable to recall their whereabouts.^8^ Launched on March 20, the Trace Together application was developed to facilitate contact tracing by monitoring users’ locations and alerting any user who has come into contact with any individual who has tested positive for COVID-19.^11^ In addition to promoting effectively sequestering individuals at high risk of infecting others, the data from contact tracing provides lead time for medical service planners and staff to prepare, and thus reduce the chance of completely overwhelming the healthcare system.^9^

Following the outbreak of the virus, those in contact with clusters or returning from overseas were presented with Stay-Home Notices (SHNs) requiring that they remain in a designated residence for a total of 14 days to minimize contact with others in order to monitor for symptoms.^12,13^ In the initial phases of the SHN policy, many government-owned chalets and university hostels were made available as quarantine facilities in order to increase adherence to SHNs.^1^ To further increase compliance, those on SHN are required to give updates on their location by using the GPS on their phones or taking pictures of their surroundings and are also subject to random house visits or phone calls from authorities.^1^ Harsh measures have been implemented in order to increase adherence to SHN; failure to comply with SHN on the first offense can result in a fine not exceeding S$10,000 or imprisonment for a term not exceeding 6 months, or both.^12^ In an effort to accommodate a surge of incoming Singaporeans returning from other countries, the government engaged various hotels to house those without other options to serve the 2-week SHN period; as of June 15, there has been only one imported case since May 10.^14^

Physical distancing measures became increasingly stringent over time in accordance with the number of cases emerging in Singapore. When the Disease Outbreak Response System Condition (DORSCON) level was raised to orange on February 7, large-scale events, regular temperature-taking, and suspension of extra-curricular activities in schools were implemented.^15^ On March 24, the Singapore government introduced stricter limitations on gatherings including the closing of entertainment venues, physical distancing measures within malls and restaurants, and the suspension of religious services.^16^ Supplementing these physical distancing measures are newly implemented physical distancing procedures, in which people who do not remain at least one meter apart or meet in groups of more than ten individuals could be subject to six months in prison or a S$10,000 fine.^17^ The timeline for the progression of COVID-19 and policy actions taken in Singapore to respond to COVID-19 are shown in Figure 1.

In an effort to reduce crowding on public transport and curb community spread, the Singaporean government announced the beginning of a “circuit breaker” period beginning on April 7 and lasting until June 1.^18^ Non-essential workplaces and schools were closed, and gatherings between individuals who do not share the same household in both public and private spaces were effectively prohibited.^18^ In addition, although the Singaporean government had initially followed the WHO’s recommendations regarding mask-wearing, it reversed its stance on April 14 by requiring mask-wearing for all public outings, regardless of duration.^19^ In order to provide access to masks, reusable masks were distributed to all households.^18^ Greater efforts to increase physical distancing in the community as of 21 April have resulted in the allocation of grocery shopping hours, and the reduction of services considered essential and therefore able to operate.^20^ Although numbers have been climbing, these numbers have mainly been attributed to the cases involving foreign workers, and cases of COVID-19 within the community have stayed about the same.^21^

In accordance with the evolving situation, Singapore has continued to use harsh penalties as a method of maintaining compliance to circuit breaker regulations. In conjunction with the aforementioned Infectious Disease Act, the COVID-19 Temporary Measures Act was passed on April 7, putting aforementioned circuit breaker measures into law.^22^ The bill also states that a one-meter distance must be kept between individuals in public spaces.^22^ Although enforcement officers initially issued written warnings to first-time offenders who defied circuit breaker rules when the circuit breaker began on April 7, these warnings quickly evolved into $300 fines for first-time offenders on April 11.^23^ Repeat offenders have faced higher penalties.^23^

Vigilance in apprehending those who flout the rules has resulted in the government recruiting about 3,000 people as safety ambassadors and enforcement officials in the community in order to enforce circuit breaker rules.^24^ Because approximately 20% of the population are still traveling for essential work, the role of these enforcement officials is to encourage physical distancing and discourage crowds from gathering in public spaces.^19^ Those who are caught violating circuit breaker measures can be charged under the Infectious Disease Act.^24^ In addition, an app called OneService has been developed which allows for ordinary citizens to report others who witness flouting of circuit breaker rules; the mobile app is yet another instrument for the Singaporean government to ensure that Singaporeans within the community are adhering to physical distancing measures.^25^

Although the first case of a foreign worker contracting COVID-19 was reported on February 8, foreign workers were initially deemed an essential service, and were permitted to continue working.^26^ As many dormitories that house foreign workers became hotspots for the transmission of COVID19, the government moved quickly to designate the dormitories as isolation areas.^27^  Authorities have been working to move workers out of the congested dormitories into empty public housing units or military camps to ensure that physical distancing can be achieved with greater ease.^21^ As of April 20, approximately 180 thousand foreign workers have been issued stay-home notices in order to isolate those who may be contagious and reduce the burden on the healthcare system.^28,29^ Within the dormitories, those who test positive are immediately isolated from those who test negative, and the government has continued to pay for both the wages and medical expenses of the foreign workers.^29^

Following the end of the circuit breaker on June 1, Singapore began a staged reopening of the country in three phases.^30^ Upon the beginning of Phase 1 on June 2, a selection of workplaces resumed activity, schools reopened, and visitation of grandparents or parents was permitted.^30^ The beginning of Phase 2 has yet to be announced as of June 15, but it will entail the opening of retail outlets, public facilities, and allow for social gatherings of up to 5 persons.^30^ Until the development of a vaccine, Singapore will remain in Phase 3, which entails the recommencement of social, cultural and business gatherings.^30^

**S2 Appendix: COVID-19 cases in Singapore**

As of June 12, 39,850 confirmed cases of COVID-19 have been reported in Singapore, of which 39,270 are locally transmitted and 580 imported. Of these, 28,040 have fully recovered and have been discharged from various hospitals and community-based facilities.^31^ Most of the remaining cases are stable or improving, with 239 remaining in hospitals, and 11,546 quarantined in community-based facilities. 2 are in critical condition warded in intensive care units (ICUs) at various hospitals.^31^ 25 persons have died from complications due to COVID-19.^30^ 8 persons have died from other conditions who were also infected with Covid-19.

A vast majority of local cases are clustered within the local foreign worker population. Among all the local cases, 37,488 (94.07%) are from foreign workers living in dormitories, 596 (1.52%) are from work permit, work pass or student pass holders, and the remaining 1,186 (3.02%) are Singaporean and Singapore Permanent Residents.

Dormitories are sites that are specifically built for foreign workers and are located mostly in the Northern and Western regions of Singapore. There are large scale dormitories located in individual sites, and smaller scale dormitories located at construction and other factory sites. The former is categorized under local cases as ‘foreign workers living in dormitories’ above, while the latter is falls under the ‘Work permit/work pass’ holders category.

Other locally transmitted cases have been identified to come from several clusters around Singapore, including from the start of the outbreak, churches, a mosque, a recreation centre, pre-schools, shopping malls, a gym, a wedding studio, nursing homes. Most of the imported cases, a majority of which arrived during the start of the outbreak, came from both Singaporean and non-Singaporean individuals who had travelled to China, ASEAN, countries in Asia, Western Europe and the United States.

Where publicly reported ages are last available, as of April 19, the average age of all the cases is 36.0 years old. Individuals aged 30-39 years’ account for the most of the cases (38.71%) while those aged 20-29 years and 40-49 years represents 30.36% and 17.38% of the total cases, respectively. In terms of gender breakdown, as of April 19, 5817 (88.3%) were male and 719 (10.91 %) were female. The majority of cases since then have been male because almost all foreign workers living in dormitories are male.

As of April 19, among the confirmed cases who had been quarantined in hospital and discharged, the average number of days spent at the hospital was 15.1 days. The age group that spent the highest average number of days at the hospital was the cohort aged 70-79 years (18.73 days), followed by those aged 60-69 years (17.66), and 20-29 years (15.76 days). Most of those discharged had spent fewer than 20 days (n=616, 75.03%) at the hospital.

**S3 Appendix:** Simulation results of local population

**S4 Appendix:** Simulation results migrant workers

**S5 Appendix:** Sensitivity analysis for Singapore Intervention

**S6 Appendix:** Sensitivity analysis for mitigation intervention with low isolation rate

**S7 Appendix:** Sensitivity analysis for mitigation intervention with moderate isolation rate

**S8 Appendix: Immunity assumption**

One of the counterfactual analysis undertaken was to explore the impact of immunity on the scenarios explored. To implement the immunity assumption, the model was modified as follows:

1. Individuals who recover from COVID-19 infection are assumed to become susceptible to new infection after the expiration of the immunity acquired from the infection.
2. A new variable “immunity duration” (which was assumed to be 6 months) was introduced in the model, which is the delay time from recovering from COVID-19 to becoming susceptible to infection again.
3. All other parameters remain unchanged.

**S9 Appendix: Uninhibited spread/herd immunity assumption**

One of the counterfactual analysis undertaken was to explore the impact of uninhibited spread/herd immunity on the COVID-19 infection trajectory. To implement the uninhibited spread/herd immunity assumption, the model was modified as follows:

1. We assume that all diagnosed COVID-19 cases are not quarantined. However, since all diagnosed COVID-19 cases are quarantined in Singapore, we implemented this in the model by assuming that:
   1. the flow of “infected asymptomatic undiagnosed” individuals to “infected asymptomatic diagnosed” was assumed to be non-existent. Therefore, to implement the uninhibited spread/herd immunity, we assumed that no diagnosis for infected asymptomatic individuals is done; or if diagnosis is done, no diagnosed infected individual is quarantined.
   2. the flow of “infected symptomatic undiagnosed” individuals to “infected symptomatic diagnosed” is assumed to be non-existent. Therefore, to implement the uninhibited spread/herd immunity, we assumed that no diagnosis for infected symptomatic individuals is done; or if diagnosis is done, no diagnosed infected individual is quarantined.
2. The assumption above allows individuals who are infected to live in the community and continue their usual interaction with their contacts as they seem fit. Hence the spread of COVID-19 become uninhibited due to lack of quarantine.
3. Given assumption 1, individuals moving from diagnosed asymptomatic to diagnosed symptomatic, mortality from diagnosed symptomatic, recovering from diagnosed symptomatic and asymptomatic are assumed to be zero.
4. All other parameters remain unchanged.

**S10 Appendix: References**

1. Lim J. SINGAPORE'S EXPERIENCE- COVID-19. LinkedIn. https://www.linkedin.com/pulse/singapores-experience-covid-19-jeremy-lim/. Published March 21, 2020. Accessed April 21, 2020.
2. Niehus R, Salazar PMD, Taylor A, Lipsitch M. Quantifying bias of COVID-19 prevalence and severity estimates in Wuhan, China that depend on reported cases in international travelers. *medRxiv*. February 2020. doi:10.1101/2020.02.13.20022707.
3. Pung R. Investigation of three clusters of COVID-19 in Singapore ... Lancet. https://www.thelancet.com/journals/lancet/article/PIIS0140-6736(20)30528-6/fulltext. Published March 16, 2020. Accessed April 21, 2020.
4. Straits Times. Coronavirus: Timeline of events so far. The Straits Times. https://www.straitstimes.com/asia/east-asia/coronavirus-timeline-of-events-so-far. Published April 20, 2020. Accessed April 21, 2020.
5. Goh K-T, Cutter J, Heng B-H, et al. Epidemiology and Control of SARS in Singapore. *Annals Academy of Medicine Singapore*. 2006;35:301-316.
6. James L, Shindo N, Cutter J, Ma S, Chew S. Public health measures implemented during the SARS outbreak in Singapore, 2003. *Public Health*. 2006;120(1):20-26. doi:10.1016/j.puhe.2005.10.005.
7. Tan Tock Seng Hospital. New facility designed to manage outbreak on scale of Sars set to be fully operational by May. Tan Tock Seng Hospital. https://www.ttsh.com.sg/About-TTSH/TTSH-News/Pages/New-facility-designed-to-manage-outbreak-on-scale-of-Sars-set-to-be-fully-operational-by-May.aspx. Published January 22, 2019. Accessed April 21, 2020.
8. Aravindan A. 'Drop everything, scramble': Singapore's contact trackers fight coronavirus. Reuters. https://www.reuters.com/article/us-health-coronavirus-singapore-tracing/drop-everything-scramble-singapores-contact-trackers-fight-coronavirus-idUSKBN2101A7. Published March 13, 2020. Accessed April 21, 2020.
9. Vaswani K. Coronavirus: The detectives racing to contain the virus in Singapore. BBC News. https://www.bbc.com/news/world-asia-51866102. Published March 19, 2020. Accessed April 21, 2020.
10. Anderson RM, Heesterbeek H, Klinkenberg D, Hollingsworth TD. How will country-based mitigation measures influence the course of the COVID-19 epidemic? *The Lancet*. 2020;395(10228):931-934. doi:10.1016/s0140-6736(20)30567-5.
11. Holmes A. Singapore is using a high-tech surveillance app to track the coronavirus, keeping schools and businesses open. Here's how it works. Business Insider Singapore. https://www.businessinsider.sg/singapore-coronavirus-app-tracking-testing-no-shutdown-how-it-works-2020-3?r=US&IR=T. Published March 24, 2020. Accessed April 21, 2020.
12. Gov.sg. Everything you need to know about Stay-Home Notice. MCI - Gov.SG. https://www.gov.sg/article/everything-you-need-to-know-about-the-stay-home-notice. Published March 19, 2020. Accessed April 21, 2020.
13. Liang LY. Parliament: 38,000 people on coronavirus stay-home notice, figure will rise with more returnees, says Lawrence Wong. The Straits Times. https://www.straitstimes.com/politics/parliament-38000-people-on-coronavirus-stay-home-notice-figure-will-rise-with-growing. Published March 25, 2020. Accessed April 21, 2020.
14. Mokhtar F, Mookerjee I. Bloomberg.com. https://www.bloomberg.com/news/articles/2020-03-28/in-singapore-quarantine-comes-with-a-sea-view-and-room-service. Published March 28, 2020. Accessed April 21, 2020.
15. Ministry of Health. News Highlights. Ministry of Health. https://www.moh.gov.sg/news-highlights/details/risk-assessment-raised-to-dorscon-orange. Published February 7, 2020. Accessed April 21, 2020.
16. Ministry of Health. News Highlights. Ministry of Health. https://www.moh.gov.sg/news-highlights/details/tighter-measures-to-minimise-further-spread-of-covid-19. Published March 24, 2020. Accessed April 21, 2020.
17. Ministry of Health. News Highlights. Ministry of Health. https://www.moh.gov.sg/news-highlights/details/promulgation-of-regulations-under-infectious-diseases-act. Published March 26, 2020. Accessed April 21, 2020.
18. Channel News Asia. In full: PM Lee's address on extension of 'circuit breaker' measures. CNA. https://www.channelnewsasia.com/news/singapore/covid-19-pm-lee-full-speech-circuit-breaker-extended-june-1-12662056. Published April 21, 2020. Accessed April 21, 2020.
19. Min AH, Phua R. COVID-19: Compulsory to wear mask when leaving the house, says Lawrence Wong. CNA. https://www.channelnewsasia.com/news/singapore/covid19-wearing-masks-compulsory-lawrence-wong-12640828. Published April 15, 2020. Accessed April 21, 2020.
20. Tay TF. Coronavirus: Last digit of IC to determine entry to four markets; essential workforce to be cut to 15 per cent. The Straits Times. https://www.straitstimes.com/singapore/coronavirus-last-digit-of-identity-card-number-to-determine-entry-to-four-markets. Published April 21, 2020. Accessed April 21, 2020.
21. Leung H. Why Singapore Lost Control of Its Coronavirus Outbreak. Time. https://time.com/5824039/singapore-outbreak-migrant-workers/. Published April 20, 2020. Accessed April 21, 2020.
22. Singapore Ministry of Health. What you can and cannot do during the circuit breaker period. MCI - Gov.SG. https://www.gov.sg/article/what-you-can-and-cannot-do-during-the-circuit-breaker-period. Published April 11, 2020. Accessed April 21, 2020.
23. Han GY. Coronavirus: $300 fine for first-time offenders of circuit breaker measures, no more warnings. The Straits Times. https://www.straitstimes.com/singapore/coronavirus-no-more-warnings-300-fine-for-first-time-offenders-of-circuit-breaker-measures. Published April 11, 2020. Accessed April 21, 2020.
24. Iau J. Coronavirus: Ambassadors and enforcement officers to be deployed to ensure safe distancing in HDB estates. The Straits Times. https://www.straitstimes.com/singapore/health/coronavirus-ambassadors-and-enforcement-officers-to-be-deployed-to-ensure-safe. Published April 6, 2020. Accessed April 21, 2020.
25. Heng M. Coronavirus: Donning mask is necessary but warm Singapore weather can be daunting, say many residents. The Straits Times. https://www.straitstimes.com/singapore/coronavirus-donning-mask-is-necessary-but-warm-singapore-weather-can-be-daunting-say-many. Published April 16, 2020. Accessed April 21, 2020.
26. Kurohi R. Coronavirus: Bangladeshi worker, whose wife gave birth to baby boy, out of ICU after more than 2 months. The Straits Times. https://www.straitstimes.com/singapore/health/coronavirus-bangladeshi-worker-whose-wife-just-gave-birth-to-baby-boy-out-of-icu. Published April 17, 2020. Accessed April 21, 2020.
27. Iau J. Coronavirus: 7 in 10 new Covid-19 cases in Singapore linked to foreign worker dormitories. The Straits Times. https://www.straitstimes.com/singapore/health/coronavirus-7-in-10-new-covid-19-cases-in-singapore-linked-to-foreign-worker. Published April 14, 2020. Accessed April 21, 2020.
28. Yi WK. 180k foreign workers, dependants to stay home from today. The Straits Times. https://www.straitstimes.com/singapore/manpower/180k-foreign-workers-dependants-to-stay-home-from-today. Published April 20, 2020. Accessed April 21, 2020.
29. Ang P. Coronavirus: We will look after you, ministry assures foreign workers. The Straits Times. https://www.straitstimes.com/singapore/we-will-look-after-you-ministry-assures-foreign-workers. Published April 18, 2020. Accessed April 21, 2020.
30. Gov.sg. Ending circuit breaker: phased approach to resuming activities safely [Internet]. MCI - Gov.SG. 2020 [cited 2020Jun15]. Available from: https://www.gov.sg/article/ending-circuit-breaker-phased-approach-to-resuming-activities-safely
31. Ministry of Health Singapore. COVID19 Situation Report. COVID. https://covidsitrep.moh.gov.sg/. Accessed April 24, 2020.
